# Safety of Medical Cannabis in Neuropathic Chronic Pain Management

**DOI:** 10.3390/molecules26206257

**Published:** 2021-10-16

**Authors:** Alessandra Bennici, Carmen Mannucci, Fabrizio Calapai, Luigi Cardia, Ilaria Ammendolia, Sebastiano Gangemi, Gioacchino Calapai, Daniel Griscti Soler

**Affiliations:** 1 Operative Unit and School of Allergy and Clinical Immunology, Department of Clinical and Experimental Medicine, University of Messina, 98125 Messina, Italy; alessandra.bennici@polime.it (A.B.); gangemis@unime.it (S.G.); daniel.grixtisoler@polime.it (D.G.S.); 2 Department of Biomedical and Dental Sciences and Morphological and Functional Imaging, University of Messina, 98125 Messina, Italy; cmannucci@unime.it; 3 Department of Chemical, Biological, Pharmaceutical and Environmental Sciences, University of Messina, 98125 Messina, Italy; fabrizio.calapai@unime.it; 4 Department of Clinical and Experimental Medicine, University of Messina, 98125 Messina, Italy; luigi.cardia@unime.it (L.C.); ilaria.ammendolia@unime.it (I.A.)

**Keywords:** *Cannabis*, cannabinoids, neuropathic pain

## Abstract

Products derived from the plant *Cannabis sativa* are widely appreciated for their analgesic properties and are employed for the treatment of chronic neuropathic pain. Only nabiximols, a product composed of two extracts containing similar percentages of the two cannabinoids cannabidiol and delta-9-tetrahydrocannabinol, is approved by regulatory authorities for neuropathic pain and spasticity due to multiple sclerosis in many European countries and Canada. It is also included in pharmacovigilance systems monitoring the occurrence of adverse drug reactions. However, it is not the same for the great variety of other cannabis preparations widely used for medical purposes. This creates a situation characterized by insufficient knowledge of the safety of cannabis preparations and the impossibility of establishing a correct risk–benefit profile for their medical use in the treatment of chronic neuropathic pain. With the aim to explore this issue more deeply, we collected data on adverse reactions from published clinical studies reporting the use of cannabis for neuropathic relief.

## 1. Introduction

Chronic pain is a common condition characterized by pain that lasts 12 weeks or more. One in five adults in Europe, or 75 million people, suffer moderate to severe pain [1]. There are three main types of pain: neuropathic, nociceptive, and nociplastic. Nociceptive pain derives from activity in neural pathways, secondary to actual tissue damage or potentially tissue-damaging stimuli. Neuropathic pain originates from lesions or dysfunction of the central or peripheral nervous system [2]. Nociplastic pain arises from altered nociception, despite no clear evidence of actual or threatened tissue damage causing the activation of peripheral nociceptors, or no evidence of disease or lesion of the somatosensory system causing the pain [3].

The plant *Cannabis sativa* has been appreciated for its medicinal properties, and its medical use in Asia dates back to ancient times. Over the centuries, however, there was dwindling interest in the health benefits of cannabis, which was renewed in the 1990s with the description of cannabinoid (CB) receptors and the identification of the endogenous cannabinoid system [4]. *Cannabis sativa* has more than 60 oxygen-containing aromatic hydrocarbon compounds, known as cannabinoids. Most of their effects seem to be mediated through cannabinoid receptors, two types of which have been isolated and cloned, CB1 and CB2. CB1 receptors are distributed widely in the nervous system and seems to have a general role in the inhibition of neurotransmitter release, whereas CB2 receptors are mainly found on cells of the immune system [5]. The most known cannabinoids are delta-9-tetrahydrocannabinol (THC) and cannabidiol (CBD). THC acts as a psychotomimetic agent and is responsible for most of the adverse effects (AEs) associated with the use of cannabis. CBD is not psychotomimetic, while it seems to counteract the negative effects of THC [6]. A very high binding affinity of THC with the CB1 receptor appears to mediate its effects. CBD has little binding affinity for either CB1 or CB2 receptors, but is capable of antagonizing them in the presence of THC. In fact, CBD behaves as a non-competitive negative allosteric modulator of CB1 receptor and reduces the efficacy and potency of THC [7].

Neuropathic pain (NP) is typically characterized by positive (gain of somatosensory function) and negative (loss of somatosensory function) sensory symptoms and signs [8]. Chronic NP can develop from either peripheral or central NP conditions. The International Association for the Study of Pain (IASP) published its latest classification of NP; the subtypes of chronic peripheral NP are as follows: trigeminal neuralgia (TN), chronic NP after peripheral nerve injury, painful polyneuropathy, post-herpetic neuralgia, and painful radiculopathy. Chronic central NP subtypes include chronic central NP associated with spinal cord injury (SCI) or brain injury, chronic central post-stroke pain, and chronic central NP associated with multiple sclerosis (MS) [9]. There is no single diagnostic test or pathognomonic symptom to identify NP; hence, clinical acumen is required. In this light, neuropathic screening tools have been developed as diagnostic aids, including the Leeds Assessment of Neuropathic Symptoms and Signs (LANSS), the Neuropathic Pain Questionnaire (NPQ), Douleur Neuropathique 4 Questions, and painDETECT [10]. Nabiximols (marketed as Sativex), composed of two extracts containing similar parts of CBD and THC extracts, is a cannabis-derived drug approved for neuropathic pain and spasticity due to multiple sclerosis in many European countries and Canada [11]. Authorized by regulatory agencies in different countries, Sativex is included in pharmacovigilance systems monitoring the occurrence of adverse drug reactions. The regulations are not the same for the great variety of cannabis preparations widely used for medical purposes, which has led to a situation where there is insufficient knowledge of the safety of cannabis and it is impossible to establish a correct risk/benefit profile for its medical use. Cannabis preparations that are not licensed as drugs are mostly used for pain [12].

It has been observed that plant-derived cannabinoids act on different pain targets. Together with their activity on cannabinoid receptors, on which THC action is prevalent, these substances exert their analgesic effects by interacting with G protein-coupled receptor (GPCR) 55 and other pharmacological targets, such as opioid and serotonin receptors [13,14]. CBD is also considered an inverse agonist to GPR3, GPR6, and GPR12 receptors, which are involved in the occurrence of neuropathic pain [15]. The analgesic effects of THC and CBD have been associated with the potentiation of α3 glycine receptors, which are widely diffused in the spinal cord dorsal horn and act as modulators of inflammatory pain [16]. 

With the aim of gaining an overview of the safety of cannabis use for NP relief, we selected published scientific articles describing results on efficacy and reporting the frequency and severity of adverse reactions to cannabis preparations administered for this medical purpose.

## 2. Methodology

Bibliographic research was carried out independently by two researchers (blinded to the authors) in major scientific databases (PubMed, Scopus, and Google Scholar) and a search engine of peer-reviewed literature on life sciences and biomedical topics. The investigators used the keywords “cannabis” and “pain” alone and in combination. All articles written in the English language and published in peer-reviewed scientific journals describing clinical trials and applications of cannabis extracts (CE) in oral or inhaled form for chronic neuropathic pain relief were collected and discussed. According to the PRISMA statement, PICOS (population, intervention, comparison, outcome) elements that formed the basis of this are showed in the Table 1.

**Figure 1:**
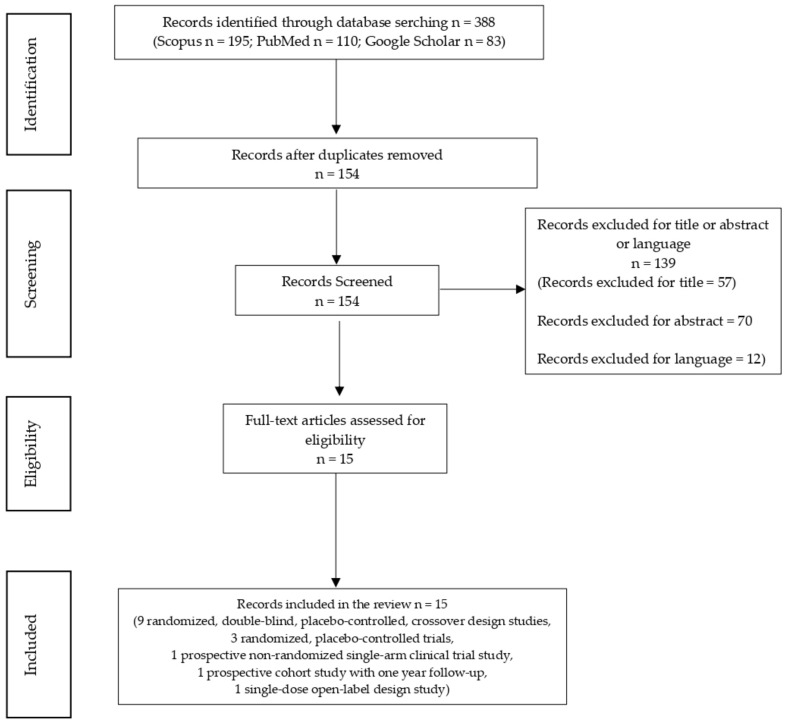
Flow chart of total records identified through database searching (*n* = 388).

The following types of scientific articles were excluded: case series, case reports, and animal studies; publications that made no reference to AEs; publications not written in the English language; studies investigating only THC or CBD individually; studies carried out using nabiximols or oromucosal/sublingual spray preparations; studies based on oncological patients; studies using co-administration of cannabis and opioids; and studies based on recreational cannabis use (Figure 1).

## 3. Results

A total of 15 articles corresponding to the same number of studies met our research criteria: 9 randomized double-blind placebo-controlled crossover studies, 3 randomized placebo-controlled trials, 1 prospective non-randomized single-arm clinical trial, 1 prospective cohort study with one year follow-up, and 1 single-dose open-label study (Figure 1). Their principal characteristics are reported in Table 2.

In the Netherlands, an experimental randomized placebo-controlled four-way crossover trial recruited 20 patients with chronic fibromyalgia pain and analyzed the analgesic effects of inhaled therapeutic cannabis. In the study, 100 mg each of three cannabis strains—Bedrocan (22.4 mg THC, 1 mg CBD), Bediol (13.4 mg THC, 17.8 mg CBD), and Bedrolite (18.4 mg CBD, 1 mg THC)—and placebo were given in a single day. There was no difference between the effects of active treatment and placebo on spontaneous pain evoked by electrical stimuli. Indeed, Bedrocan and Bediol caused a significant increase in the tolerance to pressure pain threshold. The most relevant effect was observed for the cannabis strain that contained high doses of THC and CBD (Bediol). When CBD was given with a very small dose of THC (Bedrolite, which mainly contains CBD), the analgesic effects were not superior to placebo. This result differs from those of studies in which patients with chronic pain reported beneficial effects with CBD treatment, probably related to improved anxiety, insomnia, and mood. Perhaps a single dose was insufficient to determine the analgesic effect or the dose was too low. With reference to adverse events, all three active treatments were associated with AEs related to the inhalation of cannabis, and the most common were drug high, dizziness, and nausea. There were no differences in the frequency of adverse events [17].

In Italy in 2018, a prospective non-randomized single-arm clinical trial analyzed data from 338 patients with fibromyalgia, headache, radiculopathy, and various forms of neuropathic pain. They received a daily dose of 5–40 mg/day of THC (many of the participants required 10 mg/day) as a decoction, corresponding to 28–210 mg of cannabis flos with 19% THC and 1% CBD for 12 months, and the intensity of pain was evaluated at follow-up visits. Among the patients, 33 stopped the study due to AEs, possibly due to the high percentage of THC in Bedrocan (19%), and 77 patients the study due to little benefit. The appearance of AEs was greater in the cannabis treatment groups than in the control, but they were transitory because they regressed after being interrupted. The most frequent were drowsiness and mental confusion, and other non-serious adverse events after termination of the study were worsening tachycardia, itching, diarrhea, gastralgia, nausea and vomiting, anal burning, increased depression, increased appetite, hallucinations, and muscle weakness. This study showed that treatment with cannabis is effective in reducing the intensity of pain, as measured by a visual analogue scale (VAS), and the disability caused by chronic pain as well as the resulting anxiety and depression, via the Hospital Anxiety and Depression Scale (HADS), without generating severe adverse events [18].

The safety profile of cannabis in the management of chronic non-cancerous pain was studied in a prospective cohort study, the Compass study. A 12.5% THC cannabis extract (CE) was dispensed with cannabis smoke to 215 subjects, in most cases for one year, with 216 nonsmoking subjects considered as a control group. The main outcome was the occurrence of serious adverse events (SAEs) and non-SAEs. The daily median dose was 2.5 g/d, and the recommended maximum limit was 5 g/d. No difference in the risk of SAEs between the two groups was detected, but in the cannabis group there was an increased risk of mild to moderate reactions, especially related to the nervous and psychiatric systems. The cannabis group was composed of 66% current cannabis users, 27% former cannabis users, and 6% cannabis naive users. The control group included 32% former cannabis users and 68% naive cannabis users. The most common SAEs in the cannabis group were abdominal pain, intestinal obstruction, and nephrolithiasis. However, none of the SAEs were definitely or likely related to cannabis. Two patients stopped the study due to SAEs, one with seizures considered possibly related to cannabis use and one for alcohol problems. Headache, nasopharyngitis, nausea, drowsiness, and dizziness were the most common non-SAEs reported. The cannabis group had also a higher rate of developing mild respiratory adverse events during the 12 months than the control group. In the study, the authors pointed out that cannabis users had an average decrease in FEV1 of 50 mL and an average reduction of 1% of the FEV1/FVC ratio after one year. As a secondary outcome, there was significant improvement in pain intensity and quality of life after one year for the cannabis group compared to control. In conclusion, the results of this study suggest that the adverse events with cannabis for medical use are modest and that an average dose of 2.5 g/d can be included in pain management programs safely with careful monitoring if conventional treatments have been considered inappropriate or inadequate [19].

The efficacy and tolerability of inhaled cannabis was investigated in a short-term randomized double-blind control study conducted on 16 patients with pain caused by diabetic neuropathy. Each patient was exposed to four single doses of aerosolized placebo or a low dose (1% THC), medium dose (4% THC), or high dose (7% THC) of cannabis with 1% CBD. Baseline values were collected for basic spontaneous pain, evoked pain, and cognitive tests. Pain intensity and cognitive capacity were measured for 4 h. The weight of 400 mg of plant material for administration corresponded to 0, 4, 16, or 28 mg THC per dosing session. Each participant received a placebo or a cannabis dose with 1, 4, or 7% THC with an interval of 2 weeks between doses. Adverse events were feelings of euphoria and drowsiness, significantly relevant at high and medium doses compared to placebo. This study found a dose-dependent reduction in the intensity of spontaneous and evoked pain in response to cannabis inhalation in patients with diabetic neuropathy. There were significant differences in the levels of spontaneous pain between placebo and active doses (low, medium, and high doses) and between the high dose and the other active doses. There was also impaired performance on neuropsychological tests with the high dose [20].

An Israeli study in 2014 aimed to examine the pharmacokinetics, safety, tolerability, efficacy, and ease of use of a new portable thermal dose inhaler (tMDI) for cannabis by analyzing a cohort of eight patients suffering from neuropathic pain who were on a stable analgesic regime that included medicinal cannabis. Four had complex regional pain syndrome (CRPS), two had lumbosacral radiculopathy, one had pelvic neuropathic pain, and one had pain related to spinal cord injury. Patients received a single dose of 15.1 ± 0.1 mg of cannabis through the inhaler device. A blood sample was taken to evaluate delta-9-THC and 11-hydroxy-9 THC at baseline and 120 min after inhalation of cannabis. The drug used was cannabis flos (Bedrocan) containing 19.9% THC, 0.1% CBD, and 0.2% CBN. All patients were treated with inhaled cannabis by smoking 2 or 3 times a day. The monthly median dose used was 20–30 mg. In this study, the low dose of THC produced an analgesic effect on the various conditions that caused neuropathic pain. A single inhalation containing 3.08 ± 0.02 mg of THC raised the plasma level of delta-9-THC Cmax to 38 ± 10 ng/mL and provided a 45% reduction in pain intensity [21].

In a study published in 2016 in the USA, 42 participants with neuropathic pain caused by injury or spinal cord disease were recruited, and the analgesic efficacy of 400 mg cannabis administered via the Volcano vaporizer was assessed using placebo and doses of 2.9% or 6.5% THC (11.6 mg and 26.8 mg THC, respectively). The study was carried out in three 8 h sessions with a median interval of about 7 days between sessions. The patients’ pathologies included multiple sclerosis, cervical disc pathology, spinal cord cancer, occlusion of the vertebral artery, arachnoid cysts, and syringomyelia. Among the patients, 90% had previously used cannabis.

During the session, participants inhaled 4 puffs of cannabis or placebo, and 240 min later were asked to choose a dose ranging from 4 to 8 puffs. Prior to placebo administration, there was no significant difference in the pain rate between placebo and 2.9% and 6.7% THC doses. Then there was a significant dose effect on pain intensity. The post hoc Tukey test showed a step-by-step effect with the highest pain intensity in the placebo group and the lowest in the higher THC group. Recent cannabis did not affect the results. One hour after the first treatment dose and one hour after the variable phase, both active doses were associated with significantly lower pain than placebo. Pain relief persisted for another 2 h from the variable dose, but the impact on pain showed no distinction between upper and lower doses of THC. Both active doses did not affect allodynia, which is consistent with the lack of benefit of cannabinoid treatment in postoperative pain. Many of the psychoactive side effects were concentration-dependent. The highest THC doses have been associated with significantly higher levels of “desires”, appetite, difficulty remembering things, drunkenness, and confusion [22].

A phase III multicenter clinical trial was designed to investigate a standardized oral CE used for the symptomatic relief of muscle stiffness and pain in 277 adult patients with stable MS treated with CE or placebo twice daily for 12 weeks (2 weeks titration phase, 10 weeks maintenance phase). The active treatment was an extract from *Cannabis sativa* in soft gelatin capsules containing 2.5 mg THC and 0.8–1.8 mg CBD. Subsequent doses were individually titrated upward by 5 mg THC/day every 3 days up to 12 days to optimize the ratio between therapeutic effect and side effects, and to achieve a maximum daily dose of 25 mg THC. The primary outcome was based on an 11-point category rating score (CRS) measuring perceived changes in muscle stiffness and perceived relief from body pain, muscle spasms, and sleep disturbance as a secondary outcome.

Patients treated with cannabis were divided into groups based on the severity of their symptoms of muscle stiffness and pain (low vs. high) and use of drugs (yes or no) to fight muscle spasms and pain. The level of relief, as reported by patients, was higher in the group treated with cannabis, detected after 4, 8, and 12 weeks, with the greatest difference observed in those not using antispastic drugs (37.9% cannabis vs. 16.3% placebo). When the titration period was completed, 87% of patients in the placebo group and 47% in the cannabis group took the maximum dose of 25 mg every day, and the percentages were lower at the end of the treatment period (24.5% vs. 69.4%). In the cannabis group, 33 subjects (21%) and 9 patients (6.7%) were withdrawn from or suspended treatment because of AEs. About 95% of AEs detected in all treatment groups were mild or moderate and transitory, and were not present at the end of the treatment period. SAEs were reported by 7 patients (3 patients reported urinary infections) in the cannabis group (4.9%) and 3 patients in the placebo group (2.2%). The rate of AEs was higher during the titration period in the cannabis group (75.5%). The most common AEs were related to the nervous system (71.3%) and gastrointestinal system (41.3%). AEs were more frequent in cannabis patients compared to the placebo group; they included vertigo, attention disorder, equilibrium disorder, drowsiness, dry mouth, nausea, fatigue, weakness, diarrhea, urinary infection, confusion, and falls [23].

In another publication, after randomization to placebo or smoked cannabis (4% THC), 30 participants with MS were evaluated through 8 visits over a period of 2 weeks. Patients were asked to smoke once daily for 3 days, with an 11-day washout period between treatments. Each dose was an average of 4 puffs per cigarette. The sample was composed of 63% women with an average age of 50 years old; 70% of the participants were undergoing disease-modifying therapy, and 60% were taking antispasticity agents. Most of the participants (80%) had previous recreational experience with cannabis, and 33% had used cannabis within the previous year. Those who smoked cannabis had reduced patient scores for spasticity using the modified Ashworth scale by an average of 2.74 points and on VAS by 5.28 points more than placebo. It is worth mentioning that in this study, participants began with relatively low levels of pain. Smoking cannabis did increase patient perception of “highness” by 5.04 points more than placebo. Five patients withdrew from treatment due to adverse events: two patients felt uncomfortably “high”, two had dizziness, and one had fatigue [24].

In a double-blind placebo-controlled crossover study of 39 patients with neuropathic pain, inhalation of 10.3 mg of vaporized THC, divided into 2 sessions and separated by a 2 h interval, was associated with a 31 and 25% reduction in pain intensity at 3 and 5 h, respectively. Increasing the THC dose to 28.2 mg produced an equianalgesic response that remained stable when monitored at the same time intervals (3 and 5 h). The AEs were minimal, reversible, and well tolerated. Seven patients felt a sensation of light-headedness in the first minutes after inhalation, which regressed rapidly [25].

Doses of 0, 2.5, 6, and 9.4% THC were used in a population of patients with different types of neuropathic pain. Compared to placebo, a single inhalation with a low dose, 25 ± 1 mg cannabis containing 9.4% THC, administered 3 times a day for 5 days, was associated with an average Cmax of 45 ng/mL and a decrease of 11.4% in average daily pain intensity. Adverse events were THC concentration-dependent [26].

The effects of smoked cannabis were studied in patients affected by HIV-associated distal sensory predominant polyneuropathy (DSPN) and pain resistant to other analgesic drugs in a placebo-controlled double-blind crossover trial. Participants were treated with cannabis containing 1–8% THC or placebo 4 times a day for 5 consecutive days over a period of 2 weeks. After 2 weeks of washout, each group received the other treatment. Changes in pain intensity were evaluated together with possible modifications in mood and daily functioning. Cannabis produced the greatest pain relief in comparison to placebo. Both treatments ameliorated mood and daily functioning. Two participants were withdrawn from the study due to safety issues. In particular, one subject, naïve to cannabis, showed an acute psychosis with smoked cannabis. Another participant reported a severe but transitory cough caused by cannabis. Other minor AEs were a transitory increased cardiac rate, concentration deficit, drowsiness, fatigue, more prolonged sleep duration, sense of thirst, and reduced salivation [27].

In another placebo-controlled double-blind crossover study, 38 patients with central or peripheral neuropathic pain syndrome were asked to smoke cannabis with 7% THC or 3.5% THC or a placebo during three 6 h experimental sessions. The cumulative dose at each session was 9 puffs (2 puffs after 1 h, 3 puffs after 2 h, and 4 puffs after 3 h). The primary endpoint was based on VAS pain intensity before and after smoking marijuana. Cannabis produced an analgesic effect with cumulative dosing that began to reverse within 1–2 h after the last dose. The doses of 3.5 and 7% THC were equianalgesic at every time point, with no differences between the two over time. No significant differences in outcome were observed between the different pain conditions. “Feeling high” and “feeling stoned” were more prevalent in the active treatment groups and highest in the 7% THC group. “Feeling impaired” was higher in both treatment groups, but no significant difference was found between the higher and lower THC doses. Feelings of confusion, sedation, and hunger were also higher in the two active treatment groups. No mood changes were observed. A general cognitive decline was evident in both treatment groups, with greater cognitive impairment in the high-dose group [28].

In another study, 50 patients with painful HIV-associated sensory neuropathy were assigned to smoke either cannabis with 3.57% THC or placebo cigarettes 3 times daily for 5 days. Those who smoked cannabis had reduced daily pain by 34% compared with 17% in the placebo group, and 52% of patients in the cannabis group reported >30% pain reduction compared to 24% of patients in the placebo group. No serious AEs were reported in this study. Although there were few minor AEs, side effect ratings were higher in the cannabis group than the placebo group for anxiety, sedation, disorientation, confusion, and dizziness [29].

In the Cannabinoids in Multiple Sclerosis (CAMS) study, a clinical trial of 15 weeks that recruited 630 participants, 211 participants received an oral CE, 211 received THC, and 206 received placebo. The primary outcome was the effect of cannabis on the degree of spasticity as measured by the Ashworth scale. The active pharmacological treatment was a synthetic THC capsule (Marinol) and a cannabis extract containing 2.5 mg THC, 1.25 mg cannabidiol, and <5% other cannabinoids per capsule. The dose administered was based on a scheduled titration of 5 weeks up to a maximum possible dose of 25 mg daily. In those patients with spasticity alone, cannabinoid treatment was not helpful in achieving an improved Ashworth score. However, patients who presented spasticity and pain together experienced an improvement in spasticity by 61% (*n* = 121), 60% (*n* = 108), and 46% (*n* = 91) among those who received CE, synthetic THC, and placebo, respectively. This was regarded as a subjective rather than objective clinical effect. There were reports of SAEs in all groups, but they were more frequent in the placebo group. There were also minor AEs reported: frequent episodes of dizziness, light-headedness, or dry mouth in the active groups. There were some differences between groups in gastrointestinal side effects: constipation was more frequent in the cannabis extract group, and diarrhea was reported more in the active groups and not in the placebo group. Increased appetite was also a side effect in treatment groups, although with low frequency: four in the CE group, six in the THC group, and one in the placebo group [30].

A randomized double-blind placebo-controlled twofold crossover study evaluating the safety, tolerability, and efficacy of synthetic oral THC and *Cannabis sativa* plant extract was conducted with 16 patients with MS and severe spasticity (10 with secondary progressive MS and 6 with primary progressive MS). Each patient received the following 3 treatments for 4 weeks: synthetic THC (Dronabinol), *Cannabis sativa* extract, and placebo. During the first 2 weeks, study medications (THC and extract) were administered in twice daily doses of 2.5 mg THC or plant extract containing the same level of THC. If well tolerated, the dose was elevated to 5 mg twice a day for the next 2 weeks. There was a 4 week washout period between treatments. The primary outcome was a change in VAS score for pain. All patients completed the study. Six patients had used cannabis before, none on a regular basis. Both THC and plant-extract capsules were well tolerated. No SAEs were reported. AEs were more common during plant extract treatment. Five patients reported increased spasticity during plant extract treatment. One AE was rated as severe acute psychosis lasting for 5 h after the scheduled dose increase of plant extract. No clinically relevant changes were observed on physical examination or in hematology or chemistry measurements. Because of the limited sample size, no definite conclusions were reached, but the results of this study suggested there was no therapeutic benefit with either THC or plant extract treatment. The route of administration was cited as a possible explanation for the lack of efficacy. THC is absorbed reasonably well from the gut, but the process is slow, with large variations between and within individuals. A second possible explanation could be the prescribed dose. However, even at this dose, the number of AEs, especially during plant extract treatment, was rather high, suggesting that a higher dose might not be well tolerated [31].

**Table 2:** Principal characteristics of clinical studies reporting the use of *Cannabis* for neuropathic relief.

Vaporized Administration								
Reference	Indication	Study Design and No. of Patients (pts).	Period of Treatment	Dose	Mode of Adminastration	Adverse Effects(AEs)	Serious Adverse Effects (SAEs)	Outcome
Van de Donk et al., 2019 [17]	Fibromyalgia	Randomized placebo-controlled 4-way crossover trial 25 pts	4 days	THC 22%/CBD < 1% T.D. 13.4 mg/1 mg, THC 6.3%/CBD 8% T.D. THC 13.4 mg/CBD 17.4 mg THC < 1%/CBD 9% T.D. 1 mg/18.4 mg Placebo	Vp	Sore throat, bad taste, nausea (1/3 of pts). Cough (2/3 of pts).	No	Increase in pressure pain threshold. No analgesic effect.
Wilsey et al., 2016 [22]	Spinal cord trauma or disease	Randomized, double-blind, placebo-controlled, crossover design study 42 pts	3 days	THC 2.9%, THC 6.7%, placebo minimum dose: 400 mg of cannabis.	Vp	H.D.: hungry ad memory disorders with more intensity than L.D. L.D.: high, stoned, sedated, changes in perceiving space, confused, minor attention. No withdrawal	No	Reduction in pain intensity. No significant difference in pain relief between the higher and lower dose
Wallace et al., 2015 [20]	Diabetic neuropathy	Randomized, double-blinded, placebo controlled crossover study 16 pts	4 days	THC 1%, 4%, 7% or placebo Each dose: 400 mg of cannabis	Vp	Euphoria with H.D. and M.D. Somnolence with the high dose group. No withdrawals	No	Dose-dependent reduction in the intensity of spontaneous and evoked pain.
Eisenberg et al., 2014 [21]	Complex Regional Pain Syndrome, radiculopathy, pelvic neuropathic pain, Spinal cord injury	Single-dose, open-label design 8 pts	1 day	THC 19.9%/CBD 0.1% CBN 0.2%; single dose: 15.1 mg ± 0.1 mg of cannabis flos	Vp	Lightheadedness No withdrawals	No	Effective for heterogeneous collection of neuropathic pain conditions studied.
Wilsey et al., 2013 [25]	P.N.P.: CRPS type I, diabetic neuropathy, idiopathic peripheral neuropathy, post-herpetic neuralgia, brachial plexopathy, radiculopathy. C.N.P.: spinal cord injury, Multiple Sclerosis, thalamic pain	Double-blind, placebo-controlled, crossover study 39 pts	3 days	L.D. THC (1.29%), M.D. THC (3.53%), or placebo Cannabis dose = 0.8 g of per administration.	Vp	M.D. high, stoned, sedation. Reduction of learning and memory. Light reduction during testing of psychomotor skills with the dominant hand. No withdrawals	No	Analgesic efficacy with L.D. and M.D.
**Smoked Admistration**								
**Reference**	**Indication**	**Design of the Study and No. of pts.**	**Period of Treatment**	**Dose**	**Mode of Administration**	**Adverse Effects** **(AEs)**	**Serious Adverse Effects (SAEs)**	**Outcome**
Corey-Bloom et al., 2012 [24]	Multiple Sclerosis	Randomized, double-blind, placebo controlled crossover design 37 pts	2 days	THC 4% or placebo	S	High, dizziness and fatigue. 5 participants withdrew.	No	Beneficial effects for spasticity and pain associated with multiple sclerosis.
Ware et al., 2010 [26]	Post-traumatic or post-surgical neuropathic pain	Randomized, double-blind, placebo-controlled, four period crossover design 23 pts	20 days	THC 2.5%, 6.0%, and 9.4% or placebo. Single 25-mg dose three times daily for the first five days in each cycle	S	H.D.: headache, dry eyes, burning sensation, dizziness, numbness and cough.	No	Reduction of pain intensity. Improvement of sleep, anxiety and depression.
Ellis et al., 2009 [27]	HIV-associated distal sensory predominant polyneuropathy (DSPN)	A phase II, single group, double-blind, placebo-controlled, crossover trial 34 pts	10 days	THC 1–8% or placebo Three times daily	S	Concentration difficulties, fatigue, sleepiness or sedation, increased duration of sleep, reduced salivation, thirst, increased heart rate	Induction of psychosis. Intractable cough. 2 participants were withdrawn for safety reasons.	Reduction of neuropathic pain intensity in HIV-associated DSPN
Wilsey et al., 2008 [28]	Complex regional pain syndrome type I, spinal cord injury, multiple sclerosis, peripheral neuropathy	Randomized, double-blinded, placebo-controlled, crossover design 38 pts	3 days	THC 7%, THC 3.5% or placebo	S	H.D.: impairment in attention, learning and memory, and psychomotor speed. L.D. 3.5%: decline in learning and memory. No withdrawals	No	Reduction of pain intensity. No differences were observed with the two doses.
Abrams et al., 2007 [29]	HIV-associated sensory neuropathy	Prospective randomized placebo-controlled trial 55 pts	12 days	THC 3.56% or placebo three times daily.	S	Anxiety, sedation, disorientation, paranoia, confusion, dizziness, nausea. No withdrawals	No	Relief of chronic neuropathic pain.
**Oral Adminstration**								
**Reference**	**Indication**	**Design of the Study and No. of pts.**	**Period of Treatment**	**Dose**	**Mode of Administration**	**Adverse Effects** **(AEs)**	**Serious Adverse Effects (SAEs)**	**Outcome**
Poli et al., 2018 [18]	FM, radiculopathy, headache, arthritis, various form of neuropathic pain and other chronic pain conditions	Prospective non-randomized single-arm clinical trial 338 pts	12 months	THC 19%, CBD < 1%. Starting dose 5 mg/d of THC; at 6 months dose was 10 mg/d.	D	Sleepiness and mental confusion.	No	Reduced pain intensity. Reduction in anxiety and depression.
Zajicek et al., 2012 [23]	Multiple Sclerosis (MUSEC trial)	Double blind, placebo controlled, phase III study 277 pts	12 weeks	THC 5–25 mg or placebo daily	C	Dizziness, disturbance in attention, balance disorder, somnolence, dry mouth, nausea, diarrhea, fatigue, urinary infection, disorientation. Cannabis: thirty pts withdrew due to AEs.	Urinary tract infections, head injury, and interstitial lung disease.	Reduction of muscle stiffness
Zajicek et al., 2003 [30]	Multiple sclerosis (CAMS study)	Randomised, placebo-controlled trial 630 pts	15 weeks	2.5 mg synthetic THC, 2.5 mg THC + 1.25 mg CBD, placebo. Max 25 mg THC daily	C	Dizziness, dry mouth, diarrhoea in active groups. Cannabis: constipation.	One pt died from pneumonia after 13 weeks in the THC group.	Some improvement in mobility as assessed by patients indicates a subjective clinical effect.
Killestein et al., 2002 [31]	Multiple Sclerosis	Randomized, double-blind, placebocontrolled, twofold crossover study 16 pts	12 weeks	THC, THC + 20–30% CBD, placebo. THC 2.5 mg twice daily for first two weeks, then dose was increased to 5 mg twice daily.	C	Dry mouth, headache, dizziness, increased, spasticity, somnolence, ataxia, dry mouth, emotional lability No withdrawals	Acute psychosis episode in one pt.	Results do not suggest therapeutic benefit of either THC or plant-extract treatment.
**Vaporized, Smoking, and Oral Administration**								
**Reference**	**Indication**	**Design of the Study and No. of pts.**	**Period of Treatment**	**Dose**	**Mode of Administration**	**Adverse Effects** **(AEs)**	**Serious Adverse Effects (SAEs)**	**Outcome**
Ware et al., 2015 [19]	Chronic non-cancer pain	Randomized, double-blind, placebo-controlled, four period crossover design 431 pts	12 months	THC 12.5 ± 1.5% or placebo. Median daily dose cannabis = 2.5 g/d.	S, C, and Vp	THC: headache, dry eyes, burning sensation, dizziness, numbness, cough. Cannabis: 10 pts withdrew due to AEs and 5 due to AEs and lack of efficacy.	Not statistically significant	Cannabis users: a mean 50 mL decrease in FEV1 and a mean 1% decrease in the FEV1/FVC ratio over 1 year.

THC = Tetrahydrocannabinol; CBD = cannabidiol; CBN: Cannabinol; Vp = vaporization; S = smoke; D = decoction; C = capsules; T.D. = total dose; H.D. = high dose; M.D. = medium dose; L.D. = low dose.

## 4. Quality and Risk of Bias Assessment

Low-quality clinical trials can contain errors caused by processing the results, and consequently analyzing them can lead to distorted conclusions. Quality assessment is required to prevent clinical application of inaccurate results [32]. The Risk of Bias of included RCTs was assessed according to the Cochrane RoB 2.0 (Risk of Bias 2.0), which consists of five domains and an overall judgment. The five domains are the following: (1) bias arising from the randomization process; (2) bias due to deviations from the intended interventions; (3) bias due to missing outcome data; (4) bias in measurement of the outcome; and 5) bias in selection of the reported result. Based on the answers (yes, probably yes, probably no, no, not applicable, no information) to a series of signaling questions, the judgment options within each domain consist of “low risk of bias”, “some concerns”, or “high risk of bias” [33].

According to the guidance document RoB2 recommendations, 10 of 15 studies collected [17,20,22,23,24,25,26,28,29,30] in the present article can be considered at low risk of bias. For both the two studies of Ellis et al., 2009 [27] and Killestein et al., 2002 [31], assessment of risk of bias puts in evidence a bias as a result of deviation from intended interventions. For the remaining three studies [18,19,21] assessment was not applicable (Table 3).

## 5. Discussion

The analysis of results of the studies collected in this paper shows that cannabis was found to be effective in all studies except for two early studies that were focused on multiple sclerosis patients [30,31]. In one of these, the CAMS study, only subjective improvement was reported. However, a later publication by the same authors reporting the MUSEC study, which used similar THC doses and the same capsular form and had the same primary outcome, showed a definitive improvement in muscle stiffness in multiple sclerosis with cannabis treatment. In the latter study, an 11-point numerical rating of change scale was used instead of the Ashworth scale, as the former is a more patient-oriented measure of efficacy and is now recommended by the European Medicines Agency. High placebo response rates and possible unmasking of treatment groups were cited as other plausible reasons for the negative outcome of the CAMS study. In the other study where cannabis was found to be ineffective, rather low doses of THC were used, also in capsular form. The maximum dose allowed after 4 weeks was 10 mg [31].

Medical cannabis was administered via three routes: vaporized, smoking, or oral. During the Compass Study by Ware et al., 2015, patients could choose their preferred mode of administration, with the majority opting to use a combination of smoking and vaporization together with the oral route. Those participants with a previous history of cannabis smoking chose smoking over vaporization, but half of these patients still chose a combination of smoking and oral administration. Among the participants in this study, only 11.3% had never smoked cannabis, and most of them opted for solely oral administration of the drug [19].

Most AEs reported were related to the central nervous system, and were mild and regressed rapidly. In the two 12-month-long studies included in this article, dropout rates ranged from 3–10%, with one study citing a combination of confusion and somnolence as the cause of 55% of all withdrawals [18]. One study found that AEs were more frequent during the titration period, and diminished thereafter [23]. When a median dose of 2.5 g/d of 12.5% THC was given for 1 year, corresponding to a higher dose of THC daily, the AE profile was still not remarkable and was comparable with shorter studies with lower THC doses. Psychoactive effects were generally THC dose-dependent and showed no interaction with time [25,28]. In the study by Wilsey et al., 2013 [25], sensations of any drug effect, good drug effect, feeling high, feeling stoned, and feeling sedated were more common with 3.53% THC than 1.29% THC. In a later study, the same authors found that AEs with 6.7% THC exceeded those with 2.9% THC, reporting that any drug effect, drug high, impaired, stoned, sedated, changes perceiving space, drunk, confused, and difficulty paying attention were more prevalent with the higher concentration [22], thus confirming the dose-dependence of psychoactive AEs. Wallace et al. evaluated the efficacy of inhaled cannabis in diabetic neuropathy using 1, 4, and 7% THC concentrations and placebo, and found experiences of euphoria and somnolence as the main AEs. They found that euphoria was significant with high and medium doses in contrast to placebo. On the other hand, only the high-dose cannabis had a significantly larger proportion of participants complaining of somnolence compared to placebo [20]. Countering this, a recent study by Van de Donk et al. found no difference in the frequency of AEs between the three cannabis formulations [17].

Comparing the different modes of administration of cannabis treatment, sensations of feeling high and euphoria were more prevalent in those receiving the drug via the inhalation route, with drug high reported in 40–80% of patients receiving cannabis treatment, and higher rates observed with increasing THC concentrations [17]. Despite this being seen as bothersome, it is thought that some level of intoxication may be required for an analgesic effect, with one study concluding that as the highness score increased by 1 point, the pain score decreased on average by 0.32 points [20]. Highness tended to wear off after 4 h [17,25]. Dizziness or lightheadedness had an incidence range of 10–50%. It was reported as a fleeting effect in the first minutes after inhalation of cannabis [25].

However, we noted higher rates of dizziness reported in studies on patients with multiple sclerosis who were administered cannabis treatment orally. Headaches and dry mouth were also reported more frequently in studies using the oral mode of administration, with the latter being reported in about 20% of participants receiving cannabis per os [23,30,31]. Altered bowel habits in some participants who were taking active oral treatment was reported. Diarrhea was more common in both participants taking synthetic THC and those taking CE. Constipation was much more frequent in the cannabis extract group compared to the THC group and placebo [30].

Some AEs may be directly attributable to the inhalation process, such as coughing, sore throat, and bad taste [17]. In a study carried out over a lengthy period of 12 months, residual volume was reduced among cannabis users using the inhalational route, and mild declines in FEV1 and FEV1/FVC ratio were noted [19].

Few SAEs were reported in the publications reviewed. In a study on 34 patients with HIV-associated distal sensory polyneuropathy, two subjects in the cannabis treatment group were withdrawn for safety reasons: one, who had never used cannabis before, had an acute cannabis-induced psychosis, and one developed an intractable smoking-related cough that resolved spontaneously after stopping treatment [27]. In a small study on 16 multiple sclerosis patients, one patient was reported to have an acute psychosis lasting 5 h after the scheduled dose increase of plant extract [31]. In the MUSEC trial, three SAEs, urinary tract infection (UTI), head injury, and interstitial lung disease, were considered treatment-related, with UTI accounting for 3 out of 7 SAEs in the cannabis treatment group [23]. In another study of multiple sclerosis patients, UTI was reported as an SAE across all treatment groups, with more frequent events in the placebo group [30], suggesting that this side effect could be due to the bladder dysfunction, which is common in multiple sclerosis patients.

Neurocognitive performance was often mildly impaired with cannabis treatment. In one study, 7% and 4% THC were both associated with impairment compared to placebo, but there was no significant difference between the two doses [20]. In another study comparing 7% and 3.5% THC, the higher dose resulted in impairment of attention, learning, memory, and psychomotor speed, whereas the lower dose resulted in impairment of learning and memory only, and the results in the low-dose group did not differ significantly from those in the placebo group [18]. In a 2008 study, low levels of cognitive decline were observed, affecting mostly learning and memory, with learning being inhibited by active treatment doses of 1.29 and 3.53% THC, with the higher dose scoring lower than the lower dose, and the higher dose scoring lower on memory compared to placebo [25].

In many studies, high THC concentration did not demonstrate superior analgesic effects compared to lower concentrations, except in one study investigating on a cohort of patients with diabetic neuropathy [20]. In 2016, Wilsey et al. [22] concluded that while cannabis extracts did result in reduced pain intensity in spinal cord trauma or disease, there was no significant difference in neuropathic pain relief between higher and lower THC doses. Since the psychoactive effects were dose-dependent, the authors suggested that the lower dose of 2.9% THC might be more appropriate for these patients. The same authors previously showed that in patients with central and peripheral neuropathic pain, a dose of 0.8 g of cannabis with 1.29% THC inhaled in two sessions separated by a 2 h interval was associated with a 31% and 25% reduction in pain intensity at 3 and 5 h, respectively. An increased THC concentration of 3.53% produced an equivalent analgesic response, which remained stable when monitored at the same time intervals, but with increased frequency of AEs [25]. However, this was not always the case. In a study on 16 patients with diabetic neuropathy, there was greater reduction in pain intensity in response to inhaled cannabis with the higher dose. While all active treatments performed better than placebo, there was a significant difference between the high dose (7% THC) and the other doses [20].

In terms of efficacy in the different types of disorders treated in the above selected studies, it is relevant to take into account only the 10 studies with good quality. Thus, we can observe that cannabis preparations containing 1–8% of THC administered by smoke inhalation over 10–12 consecutive days seem to produce positive effects in HIV patients with associated sensory neuropathy in the studies of Abrams et al., 2007 [29] and Ellis et al., 2009 [27]. Patients affected by complex regional pain syndrome type I, spinal cord injury, multiple sclerosis, and peripheral neuropathy who smoked cannabis starting from 3.5% THC for 3 days had reduced pain [28], and similarly for patients with post-traumatic or post-surgical neuropathic pain treated for 20 days with cigarettes containing different THC concentrations (2.5, 6.0, and 9.4%) [26]. The study by Van de Donk et al. [17] showed only a slight effect in reducing the threshold of pain in fibromyalgia by vaporization of different preparations based on combined THC and CBD.

The study by Wilsey et al., 2013 [25] investigating on several diseases with a neuropathic pain burden showed an analgesic effect of low and medium doses (1.39 and 3.53%) of vaporized THC compared with placebo. Finally, the study by Zajicek et al., 2012 [23] showed that oral administration of THC starting from a dose of 5 mg can reduce muscle stiffness in multiple sclerosis. Only one study investigating diabetic neuropathy was of good quality, and it showed positive dose-dependent effects after 4 days of vaporized THC (1%, 4%, 7%) compared to placebo [20]. Wilsey et al. (2016) [22] showed a non-dose-dependent reduction of pain with two concentrations of vaporized THC (2.9 and 6.7%) for 2 days in people with spinal cord disease or spinal trauma.

In conclusion, some forms of cannabis-derived medicinal products are available by prescription, including synthetic THC. Plant-based cannabis products contain various concentrations of the active components THC and CBD, causing unpredictability in the effects of exposure. Non-licensed plant-derived cannabis products are not assessed, as is conducted for synthetic medicinal products and natural medicinal products authorized as drugs. This situation increases the unpredictability of their potential risks to patient health [34].

The studies discussed in the present review show that cannabis is effective for short-term treatment of neuropathic pain disorders and can be considered safe, because psychotomimetic AEs are experienced by only a few patients. However, from the methodological point of view, the studies are not all of good quality. Moreover, the authors’ conclusions leave us perplexed because of the very short duration of treatment in most of them. Our view is partially in agreement with a recent systematic review investigating the efficacy, acceptability, and safety of cannabis-derived products In the analysis of data from previous systematic reviews on the treatment of chronic pain, not only neuropathic pain, sufficient evidence was not found for any chronic pain condition [35]. However, we think that results observed in the studies collected and discussed in this review are not discouraging and indicate that it is still worthwhile to continue cannabis research for the management of pain, applying more rigorous clinical methodologies and clinical designs appropriate for useful results.

Some of the more frequent AEs, include feeling high, somnolence, and confusion, which depend on the THC concentration, but are usually mild, transient, and reversible. Dizziness, headache, and nausea are also commonly reported side effects. Mild neurocognitive impairment is common. Severe adverse events are rare. Based on the current data available, the side effect profile does not change over 12 months. THC concentrations often do not differ significantly in terms of analgesic efficacy, suggesting that the lowest effective dose should be used in most cases of neuropathic pain.

There is no consistent data on the actual number of patients who experience AEs with cannabis treatment, since AEs are not always reported in a quantitative manner. In the future, it would be useful if similar studies with quantitative data specified the actual number of participants experiencing adverse events for data consistency. Moreover, most of the studies assessed in this review used the inhalation route compared to the oral route. Since there are symptoms that appear to be more frequent with one mode of administration over the other, it would be beneficial to conduct larger studies with longer duration, preferably more than 12 months, comparing the two modes to show which has a better AE profile. Finally, further limitations of the present paper are the non-homogeneous and incomplete reporting of adverse reactions and the small number of patients recruited for each study. However, a point of strength is the uniqueness of the overview, which could help clinicians in the rational use of cannabis for NP.

## Figures and Tables

**Table 1:** PICOS (population, intervention, comparison, outcome, setting) criteria for inclusion of studies.

Parameter Inclusion Criteria	
Population	Adults (≥18 years old) with chronic neuropathic pain
Intervention	Cannabis derived products
Comparison	Placebo or any other
Outcome	Improvement of pain releaf and safety of treatment
Setting	Randomized and non-randomized clinical trials

**Table 3:** Risk of Bias assessment.

Author	Bias Arising from the Randomization Process	Bias as a Result of Deviation from Intended Interventions	Bias as a Result of Missing Outcome Data	Bias in Measurement of the Outcome	Bias in Selection of the Reported Result	Overall Bias
Killestein et al., 2002 [31]	low	Some concerns	low	low	low	Some concerns
Zajicek et al., 2003 [30]	low	low	low	low	low	Low risk
Abrams et al., 2007 [29]	low	low	low	low	low	Low risk
Wilsey et al., 2008 [28]	low	low	low	low	low	Low risk
Ellis et al., 2009 [27]	low	Some concerns	low	low	low	Some concerns
Ware et al., 2010 [26]	low	low	low	low	low	Low risk
Corey-Bloom et al., 2012 [24]	low	low	low	low	low	Low risk
Zajicek et al., 2012 [23]	low	low	low	low	low	Low risk
Wilsey et al., 2013 [25]	low	low	low	low	low	Low risk
Eisenberg et al., 2014 [21]	NA	NA	NA	NA	NA	NA
Wallace et al., 2015 [20]	low	low	low	low	low	Low risk
Ware et al., 2015 [19]	NA	NA	NA	NA	NA	NA
Wilsey et al., 2016 [22]	low	low	low	low	low	Low risk
Poli et al., 2018 [18]	NA	NA	NA	NA	NA	NA
Van de Donk et al., 2019 [17]	low	low	low	low	low	Low risk

NA = Not applicable because there were not RCTs.
